# Association of Substance Use and Psychological Conditions With Adverse Pregnancy Outcomes: A Propensity-Matched Analysis

**DOI:** 10.1016/j.jacadv.2026.102805

**Published:** 2026-06-17

**Authors:** Bede N. Nriagu, Yaa Adoma Kwapong, Faith Metlock, Emine Bircan, Omotola Balogun, Sharmaine McCoy, Olayinka J. Agboola, Adhya Mehta, Antonio Saad, Jared Spitz, Vanessa Blumer, Jamie Kennedy, Lily N. Dastmalchi, Garima Sharma

**Affiliations:** a Inova Schar Heart and Vascular, Inova Health System, Falls Church, Virginia, USA; b Johns Hopkins School of Medicine, Baltimore, Maryland, USA; c University of Arkansas for Medical Sciences, Little Rock, Arkansas, USA; d Lankenau Medical Center, Main Line Health System, Wynnewood, Pennsylvania, USA; e Division of Maternal Fetal Medicine, Inova Fairfax Hospital, Falls Church, Virginia, USA

**Keywords:** adverse pregnancy outcomes, psychological conditions, substance use, Women’s health

## Abstract

**Background:**

Evidence is limited on how concurrent substance use (SU) and psychological conditions influence the risk of adverse pregnancy outcomes (APOs).

**Objectives:**

The objective of the study was to explore the association between SU and APOs among hospitalized pregnant women with psychological health conditions.

**Methods:**

A cross-sectional analysis of the National Inpatient Sample (2016-2022) identified pregnant patients hospitalized for delivery with psychological disorders (major depressive disorder, anxiety, bipolar disorder, and post-traumatic stress disorder) and SU (amphetamine/methamphetamine, cocaine, opioid, cannabis, and alcohol). Propensity scores were estimated using stepwise logistic regression, followed by 1:1 greedy matching (0.2 caliper). Logistic regression in the matched cohort estimated ORs for APOs comparing those with and without SU.

**Results:**

Among 2,040,635 weighted pregnancy- and delivery-related hospitalizations with psychological conditions, propensity score matching identified 170,745 hospitalizations with SU matched to 170,745 without SU. Compared to the group without SU, the SU group was associated with higher odds of composite APOs, adjusted OR (aOR): 1.20 (95% CI: 1.16-1.24), hypertensive disorders of pregnancy (aOR: 1.14; 95% CI: 1.09-1.18), preterm delivery (aOR: 1.51; 95% CI: 1.43-1.61), fetal growth restriction (aOR: 1.43; 95% CI: 1.35-1.51), and abruptio placenta (aOR: 1.80, 95% CI: 1.63-1.99). We observed lower odds of gestational diabetes mellitus (aOR: 0.61; 95% CI: 0.58-0.65) among the cohort with SU compared to those without.

**Conclusions:**

SU among pregnant women with psychological conditions increased the odds of most APOs (excluding gestational diabetes), highlighting the need for integrated screening and coordinated multidisciplinary care.

Substance use (SU) during pregnancy represents a significant public health challenge in the United States, with important implications for maternal and perinatal health. National data estimate that approximately 8 to 11% of pregnant women report the use of alcohol, tobacco, or illicit substances^1^ despite well-documented associations with adverse pregnancy outcomes (APOs), including hypertensive disorders of pregnancy (HDPs), preterm birth, fetal growth restriction, placental abruption, and intrauterine fetal demise.^2^, ^3^, ^4^, ^5^ The perinatal period, characterized by substantial biological and psychosocial changes, may exacerbate existing psychiatric disorders or precipitate new conditions.^6^^,^^7^

Mental health conditions are associated with an increased risk of cardiovascular disease (CVD) and APOs and are also strongly linked to a higher likelihood of SU during pregnancy.^8^, ^9^, ^10^ The persistence of SU during pregnancy is often linked to limited awareness of risks, maladaptive coping strategies, and especially coexisting psychological conditions.^11^, ^12^, ^13^ Psychological conditions themselves have become a leading contributor to preventable maternal morbidity and mortality in the United States,^14^, ^15^, ^16^, ^17^ and women with unmet mental health needs are disproportionately more likely to engage in SU.^8^^,^^9^

Despite this, prior studies have evaluated the association between SU and psychological conditions on APOs isolation, limiting understanding of how these co-occurring factors amplify risk.^2^^,^^5^^,^^16^^,^^17^ Recognizing this gap, the 2025 American Heart Association scientific statement on optimizing psychological health across the perinatal period highlighted the importance of moving beyond siloed assessment to evaluate the cumulative effects of coexisting psychosocial stressors on pregnancy outcomes.^18^

We therefore hypothesize that among pregnant women with psychological conditions, co-occurring SU would be associated with higher odds of APOs compared with psychological conditions alone. This study examines the impact of co-occurring SU and psychological health conditions on APOs among hospitalized pregnant women.

## Methods

### Data source

The National Inpatient Sample (NIS) is the largest publicly available, all-payer inpatient database in the United States, developed as part of the Healthcare Cost and Utilization Project.^19^ The NIS captures approximately 20% of hospitalizations nationwide from states representing nearly 97% of the U.S. population and includes discharge-level sampling weights that allow extrapolation to approximately 35 million hospitalizations annually in the United States.^19^ All analyses were conducted using the NIS-provided weights to generate nationally representative estimates and evaluate population-level trends.

The database includes deidentified discharge-level information on patient demographics, hospital characteristics, diagnoses, procedures, and outcomes. It has been used extensively to examine trends, associations, and outcomes of APOs among hospitalized women.^20^, ^21^, ^22^, ^23^, ^24^ As our study period began after the switch period (October 1, 2015) from the International Classification of Diseases-9th Revision, Clinical Modification to the International Classification of Diseases-10th Revision-Clinical Modification (ICD-10-CM), we utilized the ICD-10-CM codes exclusively for this analysis. Because the NIS contains only deidentified, publicly available data, this study was considered exempt from Institutional Review Board approval in accordance with institutional policy.

### Study population

**Figure 1:**
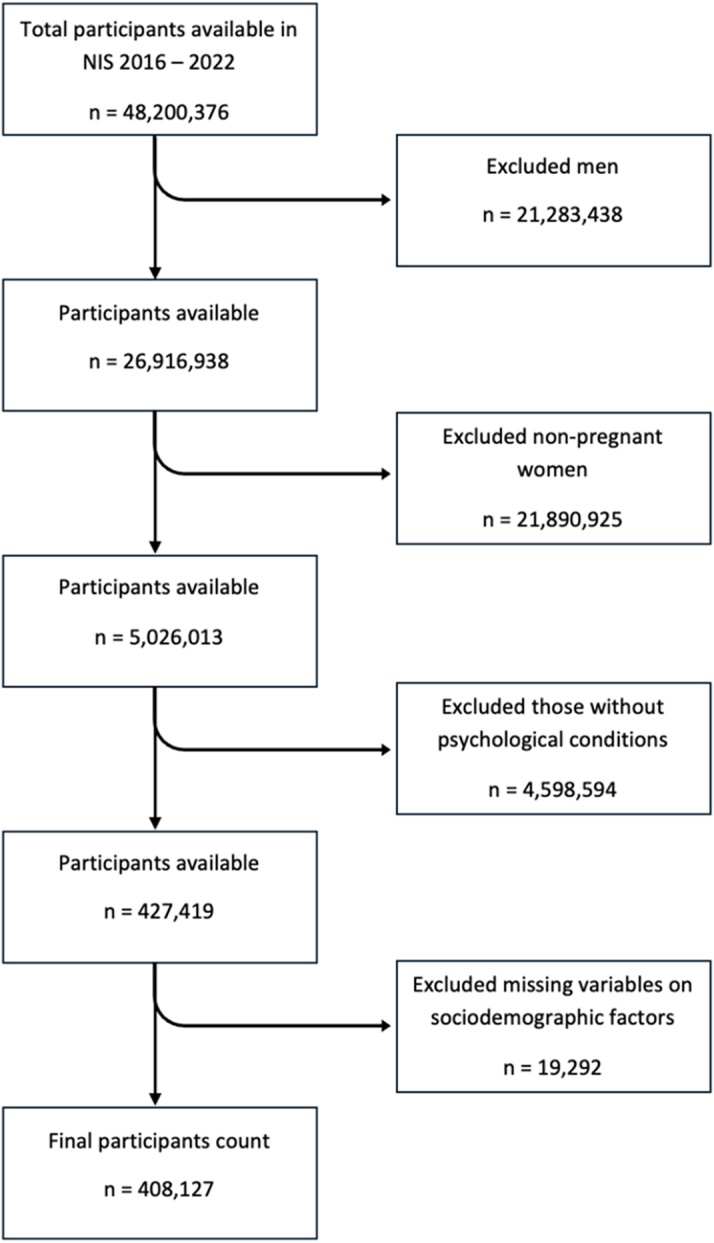
**Cohort Selection for Substance Use, Psychological Disorders, and Adverse Pregnancy Outcomes** Flow diagram illustrating selection of the analytic sample from the National Inpatient Sample (NIS) 2016 to 2022. Of 48,200,376 unweighted total hospitalizations, men were excluded, followed by nonpregnant women and those without documented psychological conditions. Hospitalizations with missing key sociodemographic data were also excluded. The final analytic cohort consisted of 408,127 unweighted pregnancy- and delivery-related hospitalizations among women with psychological conditions.

A validated definition as reported in prior studies^25^^,^^26^ was used to identify delivery-related hospitalizations among pregnant women in the NIS from January 2016 to December 2022. Psychological disturbances including major depressive disorder (F32.x–F33.x, F34.1), anxiety disorder (F40.x–F41.x, F06.4), bipolar disorder (F31.x), and post-traumatic stress disorder (PTSD) (F43.1x) were identified using ICD-10-CM code.^27^^,^^28^ Maternal SU (defined as amphetamine/methamphetamine, cocaine, opioid, cannabis, and alcohol use disorders) were identified using ICD-10-CM codes consistent with the ascertainment methods used in prior research.^29^ Tobacco use disorder was not classified as a SU in this analysis. Demographic variables (age, race/ethnicity, hospital region, hospital location/teaching status, hospital bed size, median household income by patient zip code, and primary expected payer) were further extracted, along with cardiovascular risk factors and comorbid conditions. There were 48,200,376 hospitalizations in the NIS data set 2016 to 2022. After excluding men (n = 21,283,438), nonpregnant women (n = 21,890,925), pregnant women without psychological conditions (n = 4,598,594), and records with missing sociodemographic data (n = 19,292), the final analytic sample comprised 408,127 unweighted delivery hospitalizations (Figure 1).

### Outcome

Our primary outcome was the odds of composite and individual APOs, including HDP, preterm delivery, fetal growth restriction, placental abruption, and gestational diabetes mellitus (GDM) among pregnant women with a documented history of psychological conditions. We compared those with concomitant SU to those without SU. Similar to maternal SU and psychological conditions, all hospitalizations among women with a history of APOs were abstracted using ICD-10 codes, as reported in prior studies.^30^^,^^31^ (see Supplemental Table for codes).

### Statistical analysis

**Figure 2:**
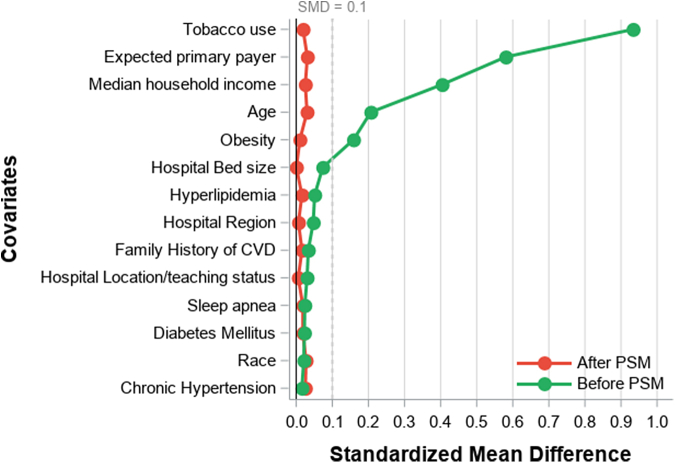
**Love Plot of Standardized Mean Differences Before and After Matching** Figure displays the balance of baseline covariates between groups before and after propensity score matching using standardized mean differences (SMDs). Each point represents a covariate, with postmatching values closer to zero indicating better balance. CVD = cardiovascular disease; PSM = propensity score matching.

Women with psychological conditions with and without concomitant SU were compared. Categorical variables were presented as counts and percentages, whereas continuous variables were presented using the mean and SD. Propensity scores for SU were estimated using survey-weighted logistic regression (PROC SURVEYLOGISTIC) incorporating NIS discharge weights, with all covariates entered simultaneously using theory-driven forced entry. Covariates were selected a priori based on established clinical and theoretical relevance to both SU and APOs, and included age, race/ethnicity, obesity, chronic hypertension, diabetes, tobacco use, hyperlipidemia, family history of CVD, sleep apnea, hospital region, hospital location and teaching status, hospital bed size, median household income quartile, and primary expected payer. One-to-one greedy nearest-neighbor matching without replacement was then performed using a caliper width of 0.2 SDs of the logit of the propensity score, yielding a balanced matched cohort of 34,149 pairs. Balance between groups was assessed using standardized mean differences (SMDs), with SMD <0.1 indicating adequate balance. A love plot was generated to visually compare SMD before and after propensity score matching (Figure 2).

Survey-weighted conditional logistic regression was performed on the propensity score-matched cohort to estimate adjusted ORs for APOs, incorporating a combined analytic weight (product of the NIS discharge and propensity score matching weights) to account for both the complex survey design and matching, as described by DuGoff et al.,^32^ to obtain unbiased, generalizable treatment effect estimates; variance was estimated using Taylor series linearization. Matched pairs were treated as strata to directly account for within-pair correlation introduced by propensity score matching. Hospital-level clustering was not incorporated, as specifying hospital as the clustering unit excluded approximately 50% of cross-hospital matched pairs. Postmatching covariate adjustment was additionally applied to address residual confounding. Models were adjusted for relevant comorbidities, including cardiomyopathy, peripartum cardiomyopathy, heart failure, ischemic heart disease, arrhythmias, valvular heart disease, stroke, pulmonary embolism, deep vein thrombosis, chronic kidney disease, congenital heart disease, prior stroke, prior cesarean delivery, and chronic anemia. Multiplicative interaction between SU and each psychiatric condition was formally tested by including a product term (SU × psychiatric condition) in survey-weighted logistic regression models, with statistical significance defined at *P* < 0.05.

Temporal trends in APOs were assessed using restricted cubic spline regression, with knots placed a priori at 2018 and 2020 to capture prepandemic changes and the onset of the COVID-19 pandemic. Nonlinearity was evaluated using a joint F-test of the spline terms. A continuous year variable estimated the overall annual change in odds (OR per year), with a categorical year model (2016 reference) as sensitivity analysis. All models incorporated survey weights to account for the complex sampling design and were conducted in the overall cohort and stratified subgroups with and without SU. Statistical analyses were conducted using SAS (9.4; SAS Institute Inc).

## Results

Among pregnant women with psychological conditions, there were 34,149 unweighted (170,745 weighted) hospitalizations among those with a history of SU and 373,978 unweighted (1,869,890 weighted) hospitalizations among those without a history of SU. Descriptive characteristics of the study population are summarized in Table 1. Overall, women with a history of SU were younger (mean age 28.1 vs 29.3 years), more likely to have composite APOs, be insured by Medicaid, and more likely to reside in zip codes with a median household income in the lowest quartile compared to those without a history of SU. Although the majority of both groups were White, women with SU were more likely to be Black or Native American and less likely to be Hispanic/Latino or Asian. Women with SU had a higher burden of psychological conditions, including bipolar disorder and PTSD, as well as higher rates of APOs such as preterm delivery, fetal growth restriction, and placental abruption. They also demonstrated lower rates of obesity and diabetes compared with women without SU (Table 1).

**Table 1:** Baseline Characteristics of Women With Psychological Conditions by History of Substance Use

	History of SU (n = 170,745)	No History of SU (n = 1,869,890)	*P* Value
Age, y, mean (SD)	28.1 (12.7)	29.3 (13.0)	<0.0001
Race/ethnicity, n (%)			
White	117,155 (68.6)	1,330,105 (71.1)	<0.0001
Black	29,430 (17.2)	207,010 (11.1)	
Hispanic/Latino	16,100 (9.4)	226,120 (12.1)	
Asian or Pacific Islander	1,315 (0.8)	40,260 (2.2)	
Native American	2,860 (1.7)	12,925 (0.7)	
Other	3,885 (2.3)	53,470 (2.9)	
Adverse pregnancy outcomes (APOs), n (%)			
Hypertensive disorders of pregnancy	31,770 (18.6)	353,890 (18.9)	0.1495
Gestational diabetes mellitus	9,550 (5.6)	189,110 (10.1)	<0.0001
Preterm delivery	15,345 (9.0)	95,955 (5.1)	<0.0001
Fetal growth restriction	15,395 (9.0)	86,340 (4.6)	<0.0001
Placenta abruption	5,355 (3.1)	25,615 (1.4)	<0.0001
Composite APOs	64,090 (37.5)	627,390 (33.6)	<0.0001
Psychological conditions, n (%)			
Major depressive disorder	85,810 (50.3)	946,575 (50.6)	0.1957
Anxiety disorder	101,250 (59.3)	1,263,255 (67.6)	<0.0001
Bipolar disorder	39,675 (23.2)	173,065 (9.3)	<0.0001
Post-traumatic stress disorder	20,975 (12.3)	86,025 (4.6)	<0.0001
Co-existing medical conditions, n (%)			
Cardiomyopathy	425 (0.3)	1710 (0.1)	<0.0001
Peripartum cardiomyopathy	150 (0.1)	845 (0.05)	0.0006
Heart failure	435 (0.3)	1,695 (0.1)	<0.0001
Ischemic heart disease	560 (0.3)	2,315 (0.1)	<0.0001
Arrhythmia	975 (0.6)	13,035 (0.7)	0.0069
Endocarditis	210 (0.1)	105 (0.01)	<0.0001
Pulmonary embolism	240 (0.1)	1,195 (0.06)	<0.0001
Deep venous thrombosis	595 (0.4)	8,175 (0.4)	0.0164
Bleeding disorders	20,320 (11.9)	205,935 (11.0)	<0.0001
Congenital heart disease	450 (0.3)	4,800 (0.3)	0.0572
Prior stroke	545 (0.3)	4,185 (0.2)	0.0005
Chronic anemia	42,715 (25.0)	412,575 (22.1)	<0.0001
Cardiovascular disease (CVD) risk factors, n (%)			
Sleep apnea	1,090 (0.6)	15,740 (0.8)	<0.0001
Obesity	39,280 (23.0)	560,600 (30.0)	<0.0001
Chronic hypertension	12,245 (7.2)	126,665 (6.8)	0.0052
Diabetes	3,100 (1.8)	39,845 (2.1)	0.0001
Tobacco use	81,755 (47.9)	179,630 (9.6)	<0.0001
Hyperlipidemia	990 (0.6)	19,395 (1.0)	<0.0001
Family history of CVD	4,015 (2.4)	53,940 (2.9)	<0.0001
Chronic renal disease	575 (0.3)	4,095 (0.2)	<0.0001
Hospital region (*N*, %)			
Northeast	33,195 (19.4)	351,290 (18.8)	<0.0001
Midwest	47,920 (28.1)	471,850 (25.2)	
South	54,115 (31.7)	644,970 (34.5)	
West	35,515 (20.8)	401,780 (21.5)	
Location/teaching status of hospital, n (%)			
Rural	18,305 (10.7)	154,490 (8.3)	<0.0001
Urban nonteaching	22,075 (12.9)	297,640 (15.9)	
Urban teaching	130,365 (76.4)	1,417,760 (75.8)	
Hospital bed size, n (%)			
Small	30,090 (17.6)	359,335 (19.2)	<0.0001
Medium	42,700 (25.0)	514,320 (27.5)	
Large	97,955 (57.4)	996,235 (53.3)	
Median household income for patients' zip code, n (%)			
0-25th percentile	65,100 (38.1)	454,325 (24.3)	<0.0001
26th to 50th percentile (median)	49,215 (28.8)	479,040 (25.6)	
51st to 75th percentile	37,085 (21.7)	500,305 (26.8)	
76th to 100th percentile	19,345 (11.3)	436,220 (23.3)	
Expected primary payer (medical insurance), n (%)			
Medicare	5,215 (3.1)	28,945 (1.6)	<0.0001
Medicaid	130,030 (76.2)	730,165 (39.1)	
Private insurance	29,730 (17.4)	1,032,315 (55.2)	
Self-pay	2,760 (1.6)	21,455 (1.2)	
No charge	55 (0.03)	510 (0.03)	
Other	2,955 (1.7)	56,500 (3.0)	

After propensity score matching, there were 34,149 hospitalizations among those with and without SU. All baseline characteristics achieved an excellent balance between groups (SMD < 0.1 for all variables) (Supplemental Table). The most substantial improvements in SMD postmatching were observed in hospital bed size, tobacco use, and primary payer status (Figure 2).

**Figure 3:**
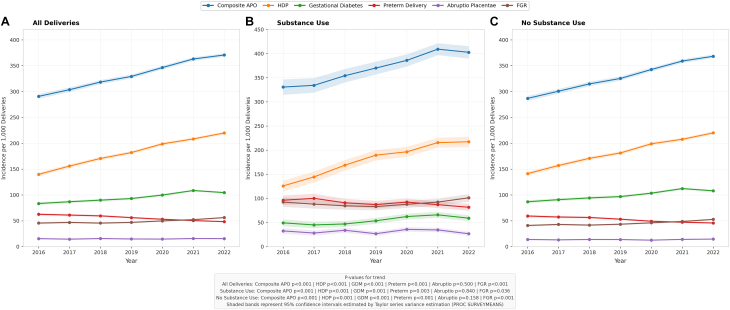
**Adverse Pregnancy Outcome Trends Among Women With Psychological Conditions, National Inpatient Sample 2016 to 2022** Annual trends in adverse pregnancy outcomes (APOs) per 1,000 deliveries among women with documented psychological conditions from 2016 to 2022, stratified by substance use (SU) status. Rates were calculated using pregnancy- and delivery-related hospitalizations for each study year. APOs = adverse pregnancy outcomes; FGR = fetal growth restriction; HDP = hypertensive disorders of pregnancy; SU = substance use.

Figure 3 shows temporal trends in APOs among women with psychological conditions (2016-2022), stratified by SU. The prevalence of composite APO increased from 290.7 per 1,000 deliveries in 2016 (54,000/185,740) to 370.7 per 1,000 in 2022 (146,850/396,155), corresponding to a 27.5% increase (OR per year: 1.08; 95% CI 1.07-1.09; non-linearity *P* = 0.037). This rise was driven by HDP which increased from 139.8 (25,965/185,740) in 2016 to 219.9 (87,105/396,155) per 1,000 in 2022 (OR: 1.11; 95% CI: 1.09-1.13; *P* < 0.001), and GDM, which increased from 83.4 (15,495/185,740) in 2016 to 104.5 (41,400/396,155) per 1,000 in 2022 (OR: 1.10; 95% CI: 1.08-1.19; *P* < 0.001). Fetal growth restriction increased modestly (45.6 [8,465/185,740] to 56.2 [22,245/396,155] per 1,000; OR: 1.01; 95% CI: 0.99-1.04; *P* = 0.001). In contrast, preterm delivery declined (OR: 0.96; 95% CI: 0.94-0.98; *P* = 0.73), whereas abruptio placentae showed no significant trend (OR: 1.02; 95% CI: 0.97-1.06; *P* = 0.31) (Table 2).

**Table 2:** Annual Incidence Rates of Adverse Pregnancy Outcomes per 1,000 Deliveries and Temporal Trend Analysis, NIS 2016–2022

	Total Deliveries	Composite APO	HDP	Gestational DM	Preterm Delivery	Abruptio Placentae	FGR
		Rate (95% CI); No. of Events	Rate (95% CI); No. of Events	Rate (95% CI); No. of Events	Rate (95% CI); No. of Events	Rate (95% CI); No. of Events	Rate (95% CI); No. of Events
Overall population							
2016	185,740	290.73 (286.11-295.35; 54,000)	139.79 (136.27-143.32; 25,965)	83.42 (80.61-86.24; 15,495)	62.56 (60.10-65.02; 11,620)	15.48 (14.22-16.73; 2,875)	45.57 (43.45-47.70; 8,465)
2017	214,065	303.60 (299.24-307.95; 64,990)	155.86 (152.43-159.30; 33,365)	86.84 (84.18-89.5; 118,590)	61.06 (58.79-63.32; 13,070)	14.46 (13.33-15.59; 3,095)	46.86 (44.85-48.86; 10,030)
2018	249,870	318.37 (314.28-322.45; 79,550)	170.63 (167.33-173.93; 42,635)	89.95 (87.44-92.46; 22,475)	59.51 (57.44-61.59; 14,870)	15.61 (14.52-16.69; 3,900)	45.54 (43.72-47.37; 11,380)
2019	286,225	329.27 (325.42-333.12; 94,245)	181.99 (178.83-185.15; 52,090)	93.02 (90.64-95.40; 26,625)	56.09 (54.21-57.98; 16,055)	14.83 (13.84-15.82; 4,245)	46.87 (45.14-48.60; 13,415)
2020	324,980	346.44 (342.78-350.09; 112,585)	198.75 (195.68-201.82; 64,590)	99.79 (97.49-102.09; 32,430)	52.96 (51.24-54.68; 17,210)	14.57 (13.65-15.49; 4,735)	49.74 (48.07-51.41; 16,165)
2021	383,600	363.03 (359.63-366.44; 139,260)	208.32 (205.44-211.19; 79,910)	108.56 (106.36-110.76; 41,645)	50.40 (48.86-51.95; 19,335)	15.64 (14.76-16.52; 6,000)	52.23 (50.65-53.80; 20,035)
2022	396,155	370.69 (367.33-374.05; 146,850)	219.88 (216.99-222.76; 87,105)	104.50 (102.37-106.63; 41,400)	48.31 (46.82-49.81; 19,140)	15.45 (14.59-16.31; 6,120)	56.15 (54.55-57.76; 22,245)
% change 2016–2022		**+27.5%**	**+57.3%**	**+25.3%**	**−22.8%**	**−0.2%**	**+23.2%**
OR per year (95% CI)		1.080 (1.067-1.093)	1.110 (1.094-1.126)	1.096 (1.075-1.118)	0.959 (0.935-0.984)	1.015 (0.969-1.064)	1.012 (0.986-1.039)
Nonlinearity *P* value		***P* = 0.037∗**	***P* < 0.001∗**	***P* < 0.001∗**	*P* = 0.729	*P* = 0.306	***P* = 0.001∗**
No substance use (*n* = 373,978)							
2016	169,005	286.77 (281.95-291.59; 48,465)	141.18 (137.47-144.89; 23,860)	86.80 (83.80-89.80; 14,670)	59.23 (56.71-61.75; 10,010)	13.79 (12.54-15.03; 2,330)	40.95 (38.83-43.06; 6,920)
2017	195,280	300.64 (296.10-305.19; 58,710)	156.95 (153.35-160.56; 30,650)	90.87 (88.02-93.72; 17,745)	57.30 (55.00-59.61; 11,190)	13.14 (12.01-14.26; 2,565)	42.89 (40.88-44.90; 8,375)
2018	227,305	314.80 (310.53-319.07; 71,555)	170.81 (167.35-174.27; 38,825)	94.21 (91.53-96.90; 21,415)	56.40 (54.28-58.52; 12,820)	13.79 (12.72-14.86; 3,135)	41.66 (39.83-43.50; 9,470)
2019	261,045	325.33 (321.31-329.35; 84,925)	181.25 (177.95-184.56; 47,315)	96.82 (94.29-99.36; 25,275)	53.09 (51.17-55.02; 13,860)	13.68 (12.68-14.67; 3,570)	43.38 (41.64-45.13; 11,325)
2020	296,435	342.62 (338.80-346.44; 101,565)	198.96 (195.75-202.18; 58,980)	103.39 (100.94-105.85; 30,650)	49.17 (47.43-50.91; 14,575)	12.53 (11.64-13.43; 3,715)	46.08 (44.39-47.77; 13,660)
2021	352,730	358.99 (355.45-362.53; 126,625)	207.68 (204.69-210.67; 73,255)	112.30 (109.97-114.63; 39,610)	47.20 (45.64-48.77; 16,650)	13.98 (13.11-14.84; 4,930)	48.72 (47.13-50.31; 17,185)
2022	368,090	368.24 (364.75-371.72; 135,545)	220.07 (217.08-223.06; 81,005)	107.98 (105.73-110.22; 39,745)	45.78 (44.27-47.29; 16,850)	14.59 (13.72-15.45; 5,370)	52.72 (51.10-54.33; 19,405)
% change 2016–2022		**+28.4%**	**+55.9%**	**+24.4%**	**−22.7%**	**+5.8%**	**+28.7%**
OR per year (95% CI)		1.079 (1.065-1.092)	1.104 (1.087-1.121)	1.096 (1.074-1.118)	0.954 (0.929-0.980)	0.998 (0.949-1.050)	1.020 (0.992-1.049)
Nonlinearity *P* value		*P* = 0.116	***P* = 0.002∗**	***P* < 0.001∗**	*P* = 0.976	*P* = 0.069	***P* = 0.003∗**
Substance use (*n* = 34,149)							
2016	16,735	330.74 (314.80-346.68; 5,535)	125.78 (114.55-137.02; 2,105)	49.30 (41.96-56.63; 825)	96.21 (86.22-106.20; 1,610)	32.57 (26.55-38.58; 545)	92.32 (82.51-102.13; 1,545)
2017	18,785	334.31 (319.22-349.39; 6,280)	144.53 (133.29-155.77; 2,715)	44.98 (38.35-51.61; 845)	100.08 (90.48-109.68; 1,880)	28.21 (22.92-33.51; 530)	88.10 (79.04-97.17; 1,655)
2018	22,565	354.31 (340.35-368.27; 7,995)	168.85 (157.92-179.78; 3,810)	46.98 (40.80-53.15; 1,060)	90.85 (82.46-99.23; 2,050)	33.90 (28.62-39.18; 765)	84.64 (76.52-92.77; 1,910)
2019	25,180	370.13 (356.80-383.47; 9,320)	189.63 (178.81-200.46; 4,775)	53.61 (47.39-59.84; 1,350)	87.17 (79.38-94.96; 2,195)	26.81 (22.35-31.27; 675)	83.00 (75.38-90.62; 2,090)
2020	28,545	386.06 (373.43-398.69; 11,020)	196.53 (186.22-206.84; 5,610)	62.36 (56.09-68.63; 1,780)	92.31 (84.80-99.82; 2,635)	35.73 (30.92-40.55; 1,020)	87.76 (80.42-95.10; 2,505)
2021	30,870	409.30 (397.03-421.56; 12,635)	215.58 (205.32-225.84; 6,655)	65.92 (59.73-72.11; 2,035)	86.98 (79.95-94.01; 2,685)	34.66 (30.10-39.22; 1,070)	92.32 (85.10-99.54; 2,850)
2022	28,065	402.81 (389.98-415.65; 11,305)	217.35 (206.56-228.14; 6,100)	58.97 (52.81-65.13; 1,655)	81.60 (74.43-88.76; 2,290)	26.72 (22.50-30.94; 750)	101.19 (93.30-109.08; 2,840)
% change 2016–2022		**+21.8%**	**+72.8%**	**+19.6%**	**−15.2%**	**−18.0%**	**+9.6%**
OR per year (95% CI)		1.099 (1.055-1.146)	1.183 (1.124-1.245)	1.112 (1.017-1.215)	0.988 (0.921-1.059)	1.112 (0.989-1.250)	0.968 (0.904-1.037)
Nonlinearity *P* value		*P* = 0.214	***P* = 0.002∗**	*P* = 0.320	*P* = 0.856	*P* = 0.120	***P* = 0.012∗**

Rates presented as rate per 1,000 deliveries (95% CI) with weighted event count (*n* events) below. 95% CIs calculated using Taylor series variance estimation via PROC SURVEYMEANS. Temporal trends assessed by restricted cubic spline regression with knots placed a priori at 2018 and 2020.

Rates per 1,000 weighted deliveries with 95% CI in parentheses; n events = weighted event count (numerator). OR = OR per year from the linear component of the restricted cubic spline model. Nonlinearity *P* value = joint F-test of spline knot terms (knots at 2018 and 2020); ∗*P* < 0.05 indicates significant nonlinear trend.

APO = adverse pregnancy outcome; DM = diabetes mellitus; FGR = fetal growth restriction; HDP = hypertensive disorders of pregnancy.

Among women with psychological conditions, composite APOs increased significantly over time in both SU and non-SU groups (*P* < 0.001), with consistently higher prevalence in the SU group (Table 2). HDP increased by >50% from 2016 to 2022 in both groups (SU: 2,105/16,735 [12.6%] to 6,100/28,065 [21.7%]; non-SU: 23,860/169,005 [14.1%] to 81,005/368,090 [22.0%]). In contrast, GDM remained substantially lower in the SU group, approximately half that of the non-SU group in 2017 and 2018 (2017: 845/18,785 [4.5%] vs 17,745/195,280 [9.1%]; 2018: 1,060/22,565 [4.7%] vs 21,415/227,305 [9.4%]), corresponding to rates of 45.0 vs 90.9 and 47.0 vs 94.2 per 1,000 deliveries, respectively (Table 2). Rates of fetal growth restriction and abruptio placentae were consistently higher, about 2-fold in the SU group across all years (Table 2).

**Figure 4:**
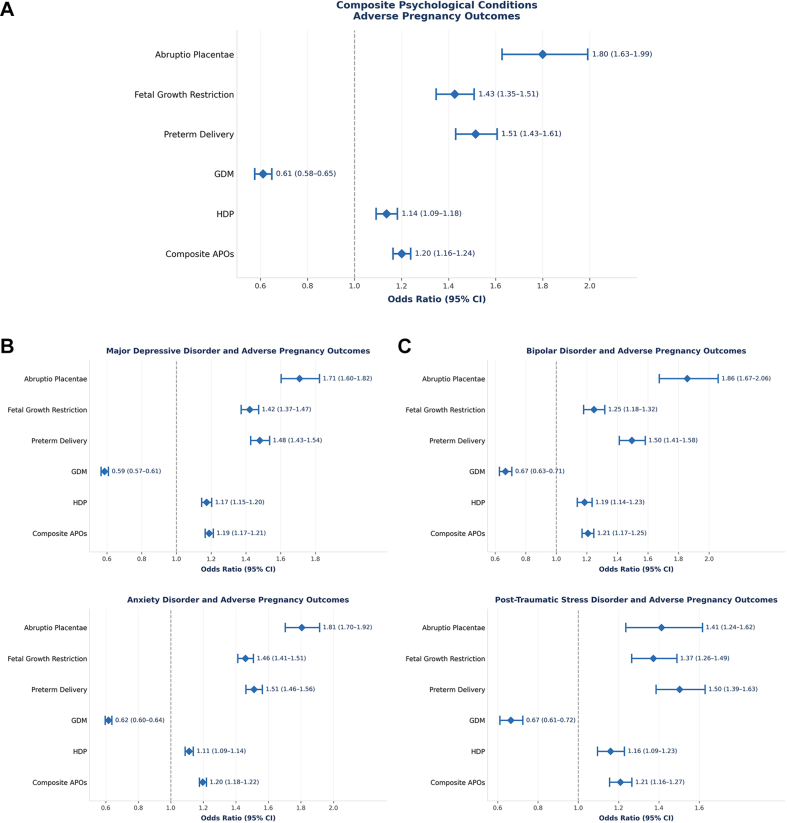
Odds of Adverse Pregnancy Outcomes in Women With Substance Use and Psychological Conditions Adjusted odds of adverse pregnancy outcomes (APOs) associated with substance use among women with coexisting psychological conditions. The figure presents adjusted odds of APOs among women with substance use and psychological conditions compared with women with psychological conditions but no history of substance use (reference group). Additional analyses stratified associations among women with major depressive disorder or anxiety disorder, and among women with bipolar disorder or post-traumatic stress disorder (PTSD). GDM = gestational diabetes mellitus; other abbreviations as in Figure 3.

Figures 4A to 4C show the adjusted odds of APOs among women with psychological conditions and SU. Overall, among women with SU, having any psychological condition was associated with 20% higher odds of experiencing any APO (adjusted OR [aOR]: 1.20; 95% CI: 1.16-1.24) compared to those without SU (Figure 4A). When examining specific outcomes, women with a history of SU and any psychological condition had 14% higher odds of HDP (aOR: 1.14; 95% CI: 1.09-1.18), 51% higher odds of preterm delivery (aOR: 1.51; 95% CI: 1.43-1.61), 43% higher odds of fetal growth restriction (aOR: 1.43; 95% CI: 1.35-1.51), and 80% higher odds of abruption of placenta (aOR: 1.80; 95% CI: 1.63-1.99) compared to those without a history of SU (Figure 4A). In contrast, the odds of GDM were 39% lower (aOR: 0.61; 95% CI: 0.58-0.65) in women with psychological conditions and concomitant SU compared to those without SU (Figure 4A).

**Central Illustration:**
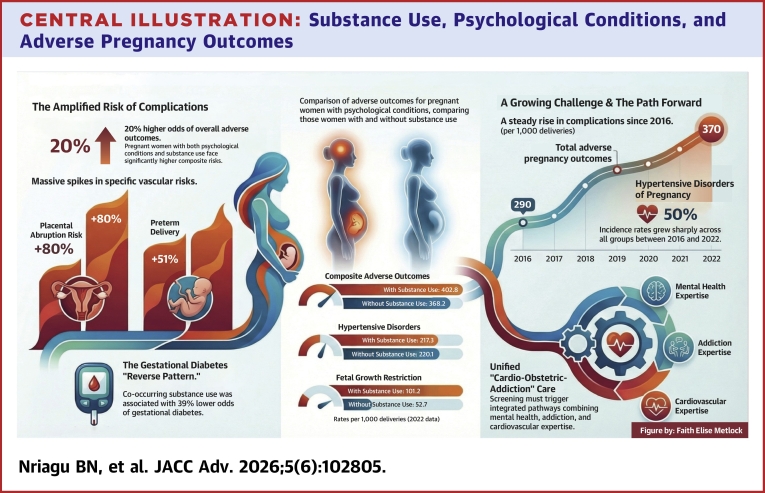
**Substance Use, Psychological Conditions, and Adverse Pregnancy Outcomes** This central illustration highlights the association of concurrent substance use (SU) on adverse pregnancy outcomes (APOs) among pregnant women with psychological conditions in a propensity score-matched cohort. Women with both psychological conditions and SU experienced significantly higher rates of composite APOs compared with those without SU. The figure compares the incidence of composite APOs, hypertensive disorders of pregnancy (HDP), and fetal growth restriction between groups and depicts temporal trends from 2016 to 2022 in the overall population with psychological conditions. Composite APOs increased steadily over the study period, driven primarily by HDP, which rose by more than 50%.

Across psychiatric subgroups, SU was consistently associated with higher odds of preterm delivery, fetal growth restriction, abruptio placentae, and HDP, and lower odds of GDM (Figures 4B and 4C). Bipolar disorder modified SU associations with HDP (amplified), GDM (attenuated), and fetal growth restriction (attenuated); PTSD modified associations with abruptio placentae and GDM (both attenuated); major depressive disorder modified associations with HDP (amplified), GDM (amplified protective), and abruptio placentae (attenuated); and anxiety disorder modified associations with HDP (attenuated) and fetal growth restriction (amplified) (all *P* < 0.05) (Supplemental Material).

## Discussion

In this nationally representative sample, we observed that women with any psychological health condition, when comorbid with SU, experienced significantly higher odds of APOs. Notably, the coexistence of mental health disorders and SU was associated with markedly elevated risks of preterm birth, HDP, fetal growth restriction, and placental abruption, suggesting a compounded burden of psychosocial stressors during pregnancy. However, we did not observe a higher odds of GDM among women with psychological conditions and SU.

Temporal trends reveal a steady rise in APOs among women with and without SU and psychological conditions in recent years. Women with concomitant SU consistently experienced a disproportionately higher burden of composite APOs compared with their non-SU counterparts. Temporal trends in APOs, from our findings, echo broader global patterns: the rising prevalence of GDM^33^ and hypertensive disorders^34^ among pregnant women. The steady increase in APOs we observed highlights the growing clinical and public health burden, particularly within populations affected by psychological comorbidities.

Our findings are consistent with prior literature demonstrating a link between maternal mental health and obstetric complications. Depression and anxiety have been reported to be independently associated with HDP,^35^^,^^36^ and preterm delivery.^35^ Prior work by McDonald et al.^37^ showed that mental illness, SU, and trauma amplify the risk of APOs when present alone or together. Our study extends this evidence by assessing the association of SU and psychiatric conditions on composite APOs as well as each APO separately, and additionally examining trends over time. The observed amplification of SU-associated HDP risk in the presence of bipolar disorder is consistent with prior reports^38^^,^^39^ of elevated gestational hypertension risk among patients with bipolar disorder, suggesting that the convergence of these 2 conditions may synergistically heighten HDP risk beyond the independent contribution of either condition alone.

Several biologic and psychosocial pathways may explain our findings. Chronic stress, adverse childhood experiences (ACEs) and trauma which are common among women with psychological disorders, are major drivers of both SU and APOs.^40^ Data from 7 states show that women with 4 or more ACEs or multiple stressful life events were significantly more likely to report postpartum SU and polysubstance use, compared to women with fewer stressors.^41^ In addition, evidence from a meta-analysis robustly links ACEs and early-life trauma to increased odds of depression and anxiety during pregnancy, which are risk pathways relevant to both SU and APO risks.^42^

Beyond behavioral associations, there is compelling neurobiological evidence that early trauma induces lasting changes in the stress-regulation system. Stress- and trauma-induced dysregulation of the hypothalamic-pituitary-adrenal axis, heightened systemic inflammation, and dysregulated vascular responses may contribute to the increased risk of APOs.^43^ Furthermore, polysubstance use, which is common in this population, compounds physiological stressors, making it challenging to disentangle the effects of individual substances. Our findings of higher odds of APOs among women with coexisting mental health disorders and SU suggest a synergistic effect with important implications for both prevention and clinical management.

GDM exhibited a reverse pattern compared with other APOs among the composite psychological condition group. Although any psychiatric conditions and SU were consistently associated with increased odds of HDP, preterm birth, and other APOs, they were not associated with increased odds of GDM. A plausible explanation could be that patients with SU tend to have lower rates of obesity^44^ which remains a risk factor for GDM.^45^, ^46^, ^47^ Conflicting evidence exists regarding the association between SU and GDM. In an Australian cohort of 539 pregnant women with mental health conditions, SU, alcohol, and smoking were not associated with elevated rates of GDM.^48^ Pan et al^49^ also found that preconception cannabis use was associated with increased GDM risk among individuals who had never used tobacco, although this association was not observed among those with prior or current tobacco use. However, our findings are consistent with studies that reported a lower risk of GDM among pregnant individuals with stimulant-related disorders (excluding cocaine),^50^ and alcohol use during pregnancy.^51^ These inconsistent findings across the literature may reflect heterogeneity in substance type, timing of exposure, and study methodology. The mechanisms remain unclear but may involve the appetite-suppressing and metabolic effects of stimulants, as well as reduced engagement with prenatal care among this group, which could potentially limit opportunities for GDM screening and diagnosis.^52^

These patterns highlight the importance of recognizing that not all APOs share the same underlying biology. Metabolic APOs, such as GDM, largely reflect disruptions in maternal metabolic regulation, particularly a maladaptive response to placental-mediated insulin resistance.^53^ By contrast, vascular APOs arise due to maternal vascular dysfunction, abnormal placental development, or impaired uteroplacental blood flow and include HDP, such as preeclampsia and gestational hypertension.^53^ Appreciating these distinct mechanisms provides a valuable framework for understanding heterogeneity in risk profiles among women with multiple psychosocial factors.

Prior studies have independently linked both SU and mental health conditions to acute cardiac morbidity during pregnancy. Parekh et al.^54^ demonstrated that mental health disorders are associated with acute adverse cardiovascular outcomes during delivery hospitalizations, whereas SU has similarly been associated with acute cardiovascular events and maternal mortality.^18^^,^^29^ Our findings extend this evidence by demonstrating the potential impact on long-term cardiac risk when these conditions co-occur. The 2025 American Heart Association Scientific Statement on optimizing psychological health during the perinatal period advocates moving beyond isolated assessment of individual psychosocial factors and instead evaluating the combined influence of multiple stressors.^18^ By examining multiple psychosocial exposures, our study identifies women with co-occurring psychological conditions and SU as a high-risk subgroup with direct implications for targeted prenatal screening and early intervention.

These findings support a targeted clinical approach. We recommend universal screening for both mental health conditions and SU at the initial prenatal visit using validated instruments. Women who screen positive for either condition should be assessed for the other, given the amplified risk of APOs demonstrated in our analysis. Positive screens should trigger structured risk-escalation pathways, with expansion of existing perinatal clinics to incorporate cardiovascular and addiction medicine expertise earlier in care or when clinically indicated (Central Illustration). To our knowledge, no published reports have described programs that formally integrate cardiovascular, obstetric, and SU care. Establishing such programs may require embedding cardio-obstetrics expertise within existing multidisciplinary team caring for pregnant individuals with SU including obstetric providers, addiction specialists, behavioral health clinicians, and social workers.^55^ These strategies align with contemporary calls to incorporate perinatal mental health within a comprehensive CVD prevention framework.^18^

### Study limitations

Several limitations should be noted. Our study relies on administrative data, which are prone to coding errors and may lack granular clinical detail, potentially introducing misclassification of SU and psychological conditions. The cross-sectional design limits causal inference, and self-reported measures of psychological conditions and SU may be affected by recall bias or under-reporting due to stigma. Moreover, ascertainment of SU at delivery preclude temporal inference and introduce potential detection bias and reverse causation. Since hospitalizations are the unit of analysis in the NIS, we were unable to track repeat hospitalizations among pregnant women who may have had more than one admission during the study period. Hospital-level clustering was not performed, as specifying hospital as the clustering unit would have excluded approximately 50% of cross-hospital matched pairs, potentially introducing selection bias and limiting representativeness; however, residual within-hospital correlation from unmeasured factors may have modestly affected estimate precision. Psychological conditions often co-occur and may impact associations with APOs. In addition, grouping all SU into a single category reflects real-world polysubstance exposure but may mask important heterogeneity. Different substance classes have distinct pathophysiologic, cardiovascular, and obstetric risk profiles, and the observed associations represent aggregate effects that may obscure substance-specific risks. Furthermore, the data set lacks information on timing, dose, or severity of use, which may differentially influence outcomes. Future studies with granular SU data are needed to characterize class-specific risks. As with all observational studies, unknown or unmeasured confounders, and heterogenous exposures may have influenced the observed associations despite adjustment and propensity score matching. Notably, tobacco was excluded from the SU category given its distinct behavioral and clinical profile and well-established independent associations with APOs.^56^ However, because smoking prevalence varies across psychiatric diagnoses,^57^ residual confounding or effect modification by concurrent tobacco use may persist and is acknowledged as a limitation. However, the NIS captures diverse populations across hospital types and regions, with a large sample size and scope that make it particularly well-suited for evaluating hospitalization outcomes and trends.

## Conclusions

Our study demonstrates that psychological health conditions, particularly when coexisting with SU, are strongly associated with APOs. Integrating mental health and addiction services into prenatal care may help reduce preventable APOs and improve long-term maternal cardiovascular health.

Perspectives **COMPETENCIES:** Among pregnant individuals with psychological disorders, concurrent substance use is associated with higher odds of APOs, including hypertensive disorders of pregnancy, preterm delivery, fetal growth restriction, and placental abruption. Because these conditions frequently coexist and may synergistically amplify risk, routine standardized screening and integrated multidisciplinary care are essential to reduce preventable maternal and fetal morbidity.

**TRANSLATIONAL IMPLICATIONS:** Future studies may evaluate substance-specific exposures and individual drug classes to identify differential risk profiles and targeted interventions. Translating these findings into practice will require overcoming fragmented care delivery systems and implementing integrated multidisciplinary models spanning obstetrics, cardiology, psychiatry, and addiction medicine to optimize outcomes in this high-risk population.

## Funding support and author disclosures

The authors have reported that they have no relationships relevant to the contents of this paper to disclose.
