# Supplementary material for: Association of Substance Use and Psychological Conditions With Adverse Pregnancy Outcomes: A Propensity-Matched Analysis
Source: JACC Adv. 2026 Jun 17;5(6):102805. doi: 10.1016/j.jacadv.2026.102805 (PMC13309302; doi:10.1016/j.jacadv.2026.102805)
Supplement: Supplemental Material [file mmc1.pdf]

# Association of Substance Use and Psychological Conditions with Adverse Pregnancy Outcomes: A Propensity-Matched Analysis

Bede N. Nriagu, MD, MPH[1]; Yaa Adoma Kwapong , MD, MPH[2]; Emine Bircan, PhD, MPH[3] ; Faith Metlock, PhD, RN[1];Omotola Balogun, MD, MPH[4]; Sharmaine McCoy, DNP, NP[1]; Olayinka J. Agboola, MD, MPH[1]; Adhya Mehta, MD[1]; Antonio Saad, MD[5], Jared Spitz, MD[1]; Vanessa Blumer, MD[1]; Jamie Kennedy, MD[1]; Lily N. Dastmalchi, DO, MA[1]; Garima Sharma, MD [1]

[1] Inova Schar Heart and Vascular, Inova Health System, Falls Church, VA, USA

[2] Johns Hopkins School of Medicine, Baltimore, MD, USA

[3] University of Arkansas for Medical Sciences, Little Rock, AR, USA

[4] Lankenau Medical Center, Main Line Health System, Wynnewood, PA, USA

[5] Division of Maternal Fetal Medicine, Inova Fairfax Hospital, Falls Church, VA, USA

**Supplemental Table 1: Baseline characteristics pre-and post-propensity score matching (PSM) of Cardiovascular Risk Factors and Socio-demographic Variables**

| Variable | Pre-match | | Post-match | | SMD pre-match | SMD post-match | % reduction | Balanced |
|---|---|---|---|---|---|---|---|---|
| | No SU n=373,978 | SU n=34,149 | No SU n=34,149 | SU n=34,149 | | | | |
| Demographics | | | | | | | | |
| Age, mean (SD) | 27.8 (5.6) | 28.0 (5.7) | 28.0 (5.6) | 28.1 (5.7) | 0.207 | 0.030 | 85.5% | Yes |
| Race, % (n) | | | | | 0.021 | 0.027 | - | Yes |
| White | 71.1% (266,021) | 68.6% (23,431) | 69.3% (23,670) | 68.6% (23,431) | | | | |
| Black | 11.1% (41,402) | 17.2% (5,886) | 17.4% (5,955) | 17.2% (5,886) | | | | |
| Hispanic | 12.1% (45,224) | 9.4% (3,220) | 9.1% (3,090) | 9.4% (3,220) | | | | |
| Asian/Pacific Islander | 2.2% (8,052) | 0.8% (263) | 0.6% (213) | 0.8% (263) | | | | |
| Native American | 0.7% (2,585) | 1.7% (572) | 1.6% (563) | 1.7% (572) | | | | |
| Other | 2.9% (10,694) | 2.3% (777) | 1.9% (658) | 2.3% (777) | | | | |
| Socioeconomic variables | | | | | | | | |
| Median household income quartile, % (n) | | | | | 0.404 | 0.025 | 93.8% | Yes |
| Quartile 1 (lowest) | 24.3% (90,865) | 38.1% (13,020) | 39.1% (13,360) | 38.1% (13,020) | | | | |
| Quartile 2 | 25.6% (95,808) | 28.8% (9,843) | 28.7% (9,789) | 28.8% (9,843) | | | | |
| Quartile 3 | 26.8% (100,061) | 21.7% (7,417) | 21.6% (7,377) | 21.7% (7,417) | | | | |
| Quartile 4 (highest) | 23.3% (87,244) | 11.3% (3,869) | 10.6% (3,623) | 11.3% (3,869) | | | | |
| Expected primary payer, % (n) | | | | | 0.581 | 0.031 | 94.7% | Yes |
| Medicare | 1.6% (5,789) | 3.1% (1,043) | 2.7% (924) | 3.1% (1,043) | | | | |

| Variable | Pre-match | | Post-match | | SMD pre-match | SMD post-match | % reduction | Balanced |
|---|---|---|---|---|---|---|---|---|
| | No SU n=373,978 | SU n=34,149 | No SU n=34,149 | SU n=34,149 | | | | |
| Medicaid | 39.1% (146,033) | 76.2% (26,006) | 77.9% (26,605) | 76.2% (26,006) | | | | |
| Private insurance | 55.2% (206,463) | 17.4% (5,946) | 16.5% (5,647) | 17.4% (5,946) | | | | |
| Self-pay | 1.1% (4,291) | 1.6% (552) | 1.3% (460) | 1.6% (552) | | | | |
| No charge | 0.03% (102) | 0.03% (11) | 0.01% (4) | 0.03% (11) | | | | |
| Other | 3.0% (11,300) | 1.7% (591) | 1.5% (509) | 1.7% (591) | | | | |
| Clinical comorbidities | | | | | | | | |
| Tobacco use, % (n) | 9.6% (35,926) | 47.9% (16,351) | 46.9% (16,029) | 47.9% (16,351) | 0.933 | 0.019 | 98.0% | Yes |
| Obesity, % (n) | 30.0% (112,120) | 23.0% (7,856) | 23.4% (8,001) | 23.0% (7,856) | 0.159 | 0.010 | 93.7% | Yes |
| Chronic hypertension, % (n) | 6.8% (25,333) | 7.2% (2,449) | 6.5% (2,232) | 7.2% (2,449) | 0.016 | 0.025 | - | Yes |
| Diabetes mellitus, % (n) | 2.1% (7,969) | 1.8% (620) | 1.6% (539) | 1.8% (620) | 0.023 | 0.018 | 21.7% | Yes |
| Hyperlipidemia, % (n) | 1.0% (3,879) | 0.6% (198) | 0.5% (159) | 0.6% (198) | 0.051 | 0.016 | 68.6% | Yes |
| Family history of CVD, % (n) | 2.9% (10,788) | 2.4% (803) | 2.1% (723) | 2.4% (803) | 0.033 | 0.016 | 51.5% | Yes |
| Sleep apnea, % (n) | 0.8% (3,148) | 0.6% (218) | 0.5% (171) | 0.6% (218) | 0.024 | 0.018 | 25.0% | Yes |
| Hospital characteristics | | | | | | | | |
| Hospital region, % (n) | | | | | 0.047 | 0.006 | 87.2% | Yes |
| Northeast | 18.8% (70,258) | 19.4% (6,639) | 19.3% (6,582) | 19.4% (6,639) | | | | |
| Midwest | 25.2% (94,370) | 28.1% (9,584) | 28.2% (9,640) | 28.1% (9,584) | | | | |
| South | 34.5% (128,994) | 31.7% (10,823) | 32.5% (11,093) | 31.7% (10,823) | | | | |
| West | 21.5% (80,356) | 20.8% (7,103) | 20.0% (6,834) | 20.8% (7,103) | | | | |

| Variable | Pre-match | | Post-match | | SMD pre-match | SMD post-match | % reduction | Balanced |
|---|---|---|---|---|---|---|---|---|
| | No SU n=373,978 | SU n=34,149 | No SU n=34,149 | SU n=34,149 | | | | |
| Hospital location/teaching status, % (n) | | | | | 0.030 | 0.006 | 80.0% | Yes |
| Rural | 8.3% (30,898) | 10.7% (3,661) | 11.0% (3,758) | 10.7% (3,661) | | | | |
| Urban non-teaching | 15.9% (59,528) | 12.9% (4,415) | 12.7% (4,347) | 12.9% (4,415) | | | | |
| Urban teaching | 75.8% (283,552) | 76.4% (26,073) | 76.3% (26,044) | 76.4% (26,073) | | | | |
| Hospital bed size, % (n) | | | | | 0.073 | 0.000 | 100.0% | Yes |
| Small | 19.2% (71,867) | 17.6% (6,018) | 17.5% (5,974) | 17.6% (6,018) | | | | |
| Medium | 27.5% (102,864) | 25.0% (8,540) | 25.2% (8,619) | 25.0% (8,540) | | | | |
| Large | 53.3% (199,247) | 57.4% (19,591) | 57.3% (19,556) | 57.4% (19,591) | | | | |

**Supplemental Table 1: Baseline characteristics pre-and post-propensity score matching (PSM) of Cardiovascular Risk Factors and Socio-demographic Variables. SU = substance use disorder. SMD = standardized mean difference. Balanced = SMD post-match <0.1. - indicates SMD remained below 0.1 with minimal or no reduction. Frequencies shown in parentheses are unweighted discharge counts from the National Inpatient Sample**

## Supplemental Table 2. Odds ratios for the effect of Substance Use by psychiatric subgroup across adverse pregnancy outcomes

| Psychiatric subgroup | APO | | HDP | | Gestational DM | | Preterm delivery | | Abruptio placentae | | FGR | |
|---|---|---|---|---|---|---|---|---|---|---|---|---|
| | **OR (95% CI)** | **p-values** | **OR (95% CI)** | **p-values** | **OR (95% CI)** | **p-values** | **OR (95% CI)** | **p-values** | **OR (95% CI)** | **p-values** | **OR (95% CI)** | **p-values** |
| **Major depressive disorder (MDD)** | | | | | | | | | | | | |
| SU with MDD | 1.188 (1.165–1.212) | **<0.001** | 1.174 (1.145–1.203) | **<0.001** | 0.587 (0.566–0.609) | **<0.001** | 1.480 (1.427–1.536) | **<0.001** | 1.709 (1.602–1.823) | **<0.001** | 1.422 (1.372–1.473) | **<0.001** |
| SU without MDD | 1.214 (1.190–1.238) | **<0.001** | 1.098 (1.071–1.126) | **<0.001** | 0.638 (0.614–0.663) | **<0.001** | 1.547 (1.491–1.604) | **<0.001** | 1.885 (1.770–2.009) | **<0.001** | 1.431 (1.379–1.484) | **<0.001** |
| **Anxiety disorder** | | | | | | | | | | | | |
| SU with anxiety | 1.197 (1.176–1.219) | **<0.001** | 1.112 (1.088–1.138) | **<0.001** | 0.617 (0.596–0.638) | **<0.001** | 1.512 (1.462–1.563) | **<0.001** | 1.806 (1.704–1.915) | **<0.001** | 1.459 (1.412–1.508) | **<0.001** |
| SU without anxiety | 1.209 (1.182–1.236) | **<0.001** | 1.180 (1.147–1.214) | **<0.001** | 0.603 (0.578–0.630) | **<0.001** | 1.521 (1.460–1.585) | **<0.001** | 1.789 (1.665–1.921) | **<0.001** | 1.376 (1.322–1.432) | **<0.001** |
| **Bipolar disorder** | | | | | | | | | | | | |
| SU with bipolar | 1.207 (1.169–1.246) | **<0.001** | 1.185 (1.137–1.234) | **<0.001** | 0.667 (0.628–0.709) | **<0.001** | 1.495 (1.413–1.582) | **<0.001** | 1.857 (1.674–2.060) | **<0.001** | 1.247 (1.179–1.318) | **<0.001** |
| SU without bipolar | 1.195 (1.176–1.214) | **<0.001** | 1.131 (1.109–1.153) | **<0.001** | 0.601 (0.583–0.619) | **<0.001** | 1.498 (1.455–1.542) | **<0.001** | 1.775 (1.688–1.867) | **<0.001** | 1.463 (1.422–1.505) | **<0.001** |
| **Post-traumatic stress disorder (PTSD)** | | | | | | | | | | | | |
| SU with PTSD | 1.209 (1.155–1.266) | **<0.001** | 1.160 (1.095–1.229) | **<0.001** | 0.666 (0.611–0.725) | **<0.001** | 1.503 (1.386–1.629) | **<0.001** | 1.413 (1.236–1.616) | **<0.001** | 1.372 (1.265–1.489) | **<0.001** |
| SU without PTSD | 1.197 (1.180–1.215) | **<0.001** | 1.134 (1.113–1.155) | **<0.001** | 0.607 (0.590–0.624) | **<0.001** | 1.504 (1.464–1.546) | **<0.001** | 1.836 (1.750–1.927) | **<0.001** | 1.427 (1.389–1.466) | **<0.001** |
| **Interaction p-values (SU × psychiatric condition)** | | | | | | | | | | | | |
| SU × MDD | p=0.134 | NS | **p=0.0002** | **Sig** | **p=0.0022** | **Sig** | p=0.097 | NS | **p=0.033** | **Sig** | p=0.809 | NS |
| SU × Anxiety | p=0.498 | NS | **p=0.0014** | **Sig** | p=0.438 | NS | p=0.823 | NS | p=0.833 | NS | **p=0.026** | **Sig** |

| Psychiatric subgroup | APO | | HDP | | Gestational DM | | Preterm delivery | | Abruptio placentae | | FGR | |
|---|---|---|---|---|---|---|---|---|---|---|---|---|
| | **OR (95% CI)** | **p-values** | **OR (95% CI)** | **p-values** | **OR (95% CI)** | **p-values** | **OR (95% CI)** | **p-values** | **OR (95% CI)** | **p-values** | **OR (95% CI)** | **p-values** |
| SU × Bipolar | p=0.573 | NS | **p=0.045** | **Sig** | **p=0.0024** | **Sig** | p=0.954 | NS | p=0.446 | NS | **p<0.001** | **Sig** |
| SU × PTSD | p=0.680 | NS | p=0.460 | NS | **p=0.045** | **Sig** | p=0.979 | NS | **p=0.0003** | **Sig** | p=0.373 | NS |

## Supplemental Table 2. Odds ratios for the effect of Substance Use by psychiatric subgroup across adverse pregnancy outcomes

Survey-weighted logistic regression · N = 68,298 matched pairs · OR = SU (1 vs 0) at each level of psychiatric condition · All models adjusted for cardiac and vascular comorbidities SU = substance use disorder. MDD = major depressive disorder. PTSD = post-traumatic stress disorder. OR = odds ratio. CI = 95% Wald confidence interval. APO = composite adverse pregnancy outcome. HDP = hypertensive disorders of pregnancy. GDM = gestational diabetes mellitus. FGR = fetal growth restriction. 'SU with' rows show the OR for SU among patients who have the psychiatric condition; 'SU without' rows show the OR for SU among patients who do not have that psychiatric condition. Interaction p-value = Wald chi-square for the SU × psychiatric condition product term. Sig = statistically significant (p<0.05); NS = not significant.

**Supplemental Table 3: Variables And ICD-10 / ICD-10-PCS / DRG Codes**

| Diagnosis / Category | ICD-10 / ICD-10-PCS / DRG Codes |
|---|---|
| **ADVERSE PREGNANCY OUTCOMES** | |
| Hypertensive Disorder of Pregnancy | O11*, O13*, O14*, O15 |
| Intrauterine Growth Restriction | O36.5 |
| Gestational Diabetes | O24.41x, O24.42x, O24.43x, O24.424, O24.414, O24.434, O24.415, O24.425, O24.435 |
| Placental Abruption | O45 |
| Preterm Delivery | O60.1 |
| **SUBSTANCE USE** | |
| Amphetamine / Methamphetamine Use | F15.10, F1512, F15120–F1519, F1520, F1522–F1599, T43621A, T43622A, T43623A, T43624, T43601A, T43602A, T43603A, T43604A, T43691A, T43692A, T43693A, T43694A |
| Cocaine Use | F1410, F1412–F1419, F1420, F1422–F1499, T405X1A, T405X2A, T405X3A, T405X4A |
| Opioid Use | F1110, F11120–F1119, F1120, F1122–F1199, T400X1A–T400X4A, T401X1A–T401X4A, T402X1A–T402X4A, T403X1A–T403X4A, T404X1A–T404X4A, T40601A–T40604A, T40691A–T40694A |
| Cannabis Use | F1210, F12120–F1219, F1220, F1221, F1222–F1299, T40711A–T40714A, T40721A–T40724A |

| | |
|---|---|
| Alcohol Use | F1010, F1012–F1019, F102, F1020, F1022–F1099, T510X1A–T510X4A, O99314, O99315 |
| **PRIMARY CARDIOVASCULAR OUTCOMES** | |
| Cardiomyopathy | I420–I422, I425–I429 |
| Peripartum Cardiomyopathy | O903 |
| Heart Failure | I501, I5020–I5023, I5030–I5033, I5040–I5043, I50810–I50813, I5082, I5083, I5089, I509 |
| Acute Myocardial Infarction | I210–I21A9 |
| Arrhythmia | I470–I479, I480–I4819, I483–I4892, I490–I499 |
| Cardiac Arrest | I462–I469 |
| Endocarditis | I330, I339, I38, I39 |
| Stroke | I600–I609, I610–I619, I620–I629, I630–I639 |
| **CARDIOVASCULAR RISK FACTORS** | |
| Obesity | E660, E6601, E6609, E668, E669, Z683–Z6845, O9921–O99215 |
| Chronic Hypertension | I10–I132, O10–O1093, O11–O119 |
| Pregestational Diabetes | E10–E109, E110–E119, E13, O240–O2433, O248–O2483 |
| Tobacco Use | F17200–F17299, Z720, O9933x |
| Hyperlipidemia | E780, E7800, E781, E782, E784, E7849, E785 |
| Family History of Cardiovascular Disease | Z824, Z8241, Z8249 |
| **PREEXISTING CONDITIONS** | |
| Chronic Renal Disease | N18x, I12, I12.0, I12.9, O102x, O103x |
| Congenital Heart Disease | Q20x–Q26x |

| | |
|---|---|
| Prior Stroke | Z8673 |
| Previous Cesarean Section | O3421x |
| Chronic Anemia | D50–D64x, O990x |
| **DELIVERY OUTCOMES** | |
| Outcome of Delivery | Z37 series (Z370–Z379) |
| Normal Delivery Codes | O80, O82, O758, O7582 |
| **DELIVERY PROCEDURE CODES (ICD-10-PCS)** | |
| Low Cesarean Delivery | 10D00Z0 |
| Mid Forceps Cesarean Delivery | 10D00Z1 |
| Low Forceps Cesarean Delivery | 10D00Z2 |
| Cesarean Delivery, Vacuum Extraction | 10D07Z3 |
| Cesarean Delivery, Internal Version | 10D07Z4 |
| Cesarean Delivery, Low Forceps | 10D07Z5 |
| Cesarean Delivery, Mid Forceps | 10D07Z6 |
| Cesarean Delivery, High Forceps | 10D07Z7 |
| Cesarean Delivery, Other | 10D07Z8 |
| Delivery, External Approach | 10E0XZZ |
| **DELIVERY DRG CODES** | |
| Cesarean w/ CC/MCC | DRG765 |
| Cesarean w/o CC/MCC | DRG766 |
| Vaginal Delivery w/ Sterilization or D&C | DRG767 |
| Vaginal Delivery w/ O.R. Procedure | DRG768 |
| Vaginal Delivery w/ Complicating Diagnoses | DRG774 |
| Vaginal Delivery w/o Complicating Diagnoses | DRG775 |
| Cesarean w/ Complicating Diagnoses | DRG783 |
| Cesarean w/o Complicating Diagnoses | DRG784 |

| | |
|---|---|
| Vaginal w/ Major Complications | DRG785 |
| Vaginal w/ Minor Complications | DRG786 |
| Cesarean w/ Major Complications | DRG787 |
| Cesarean w/ Minor Complications | DRG788 |
| **PREGNANCY WITH ABORTIVE OUTCOME - DIAGNOSES** | |
| Ectopic Pregnancy | O00, O000–O009 |
| Hydatidiform Mole | O01, O010–O011, O019 |
| Other Abnormal Products of Conception | O02, O020, O021, O028, O029 |
| Spontaneous Abortion | O03 series |
| Medical Abortion | O04 series |
| Failed Attempted Termination | O07 series |
| Complications of Ectopic or Molar Pregnancy | O08 series |
| **PREGNANCY WITH ABORTIVE OUTCOME - PROCEDURE CODES** | |
| Abortion (Open Approach) | 10A00ZZ |
| Abortion (Percutaneous) | 10A03ZZ |
| Abortion (Percutaneous Endoscopic) | 10A04ZZ |
| Abortion (Endoscopic) | 10A08ZZ |
| Vacuum Extraction | 10A07Z6 |
| Laminaria | 10A07ZW |
| Abortifacient | 10A07ZX |
| Other Natural/Artificial Approach | 10A07ZZ |

**Supplemental Table 2: Variables And ICD-10 / ICD-10-PCS / DRG Codes**
